# Supplementary material for: R2R - software to speed the depiction of aesthetic consensus RNA secondary structures
Source: BMC Bioinformatics. 2011 Jan 4;12:3. doi: 10.1186/1471-2105-12-3 (PMC3023696; doi:10.1186/1471-2105-12-3)

GOLLD

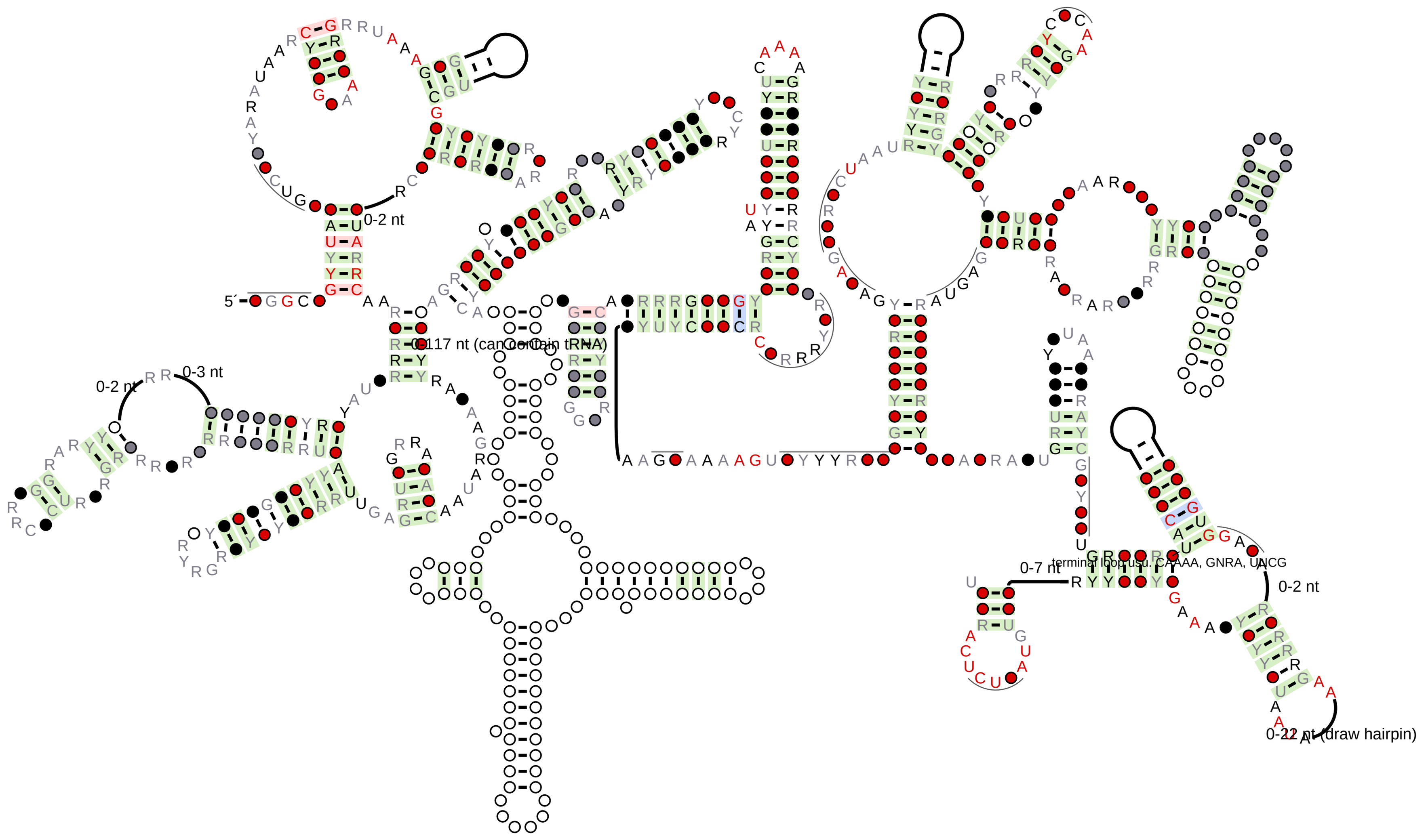

GOLLD NC_008497.1/1119234-1118463:3

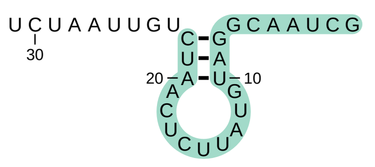

GOLLD NC_008497.1/1119234-1118463:5

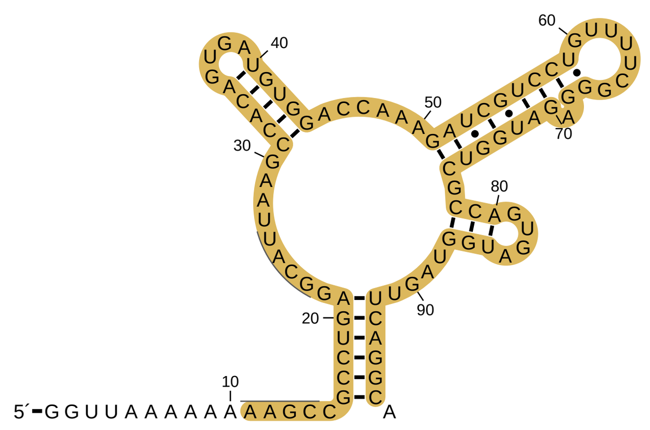

GOLLD skeleton-with-bp

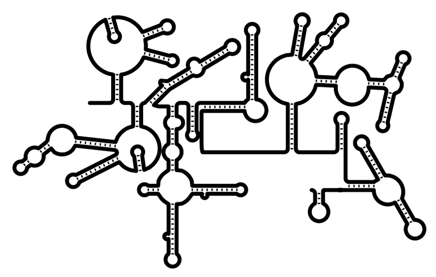

GOLLD skeleton-with-bp RACE-skeleton=1

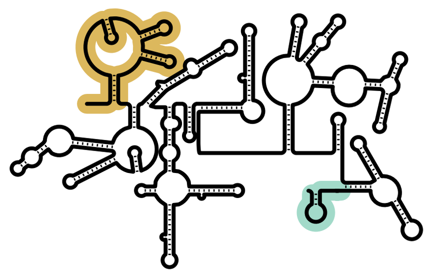

GOLLD skeleton-with-bp align-skeleton=1

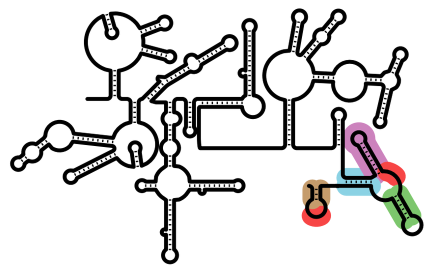

GOLLD-3vnottruncated
subfam_weight=0.716981

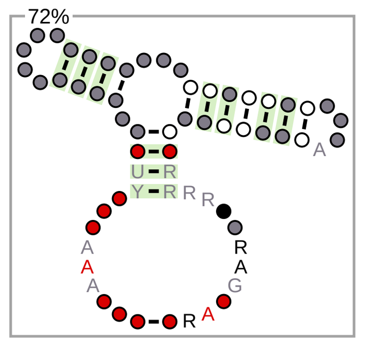

GOLLD-3vp1
subfam_weight=0.113208

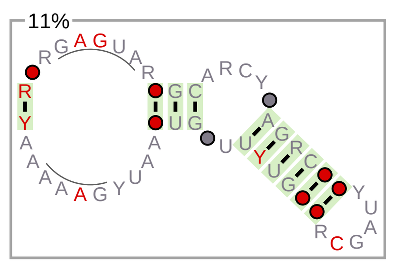

GOLLD-3vp12
subfam_weight=0.0377358

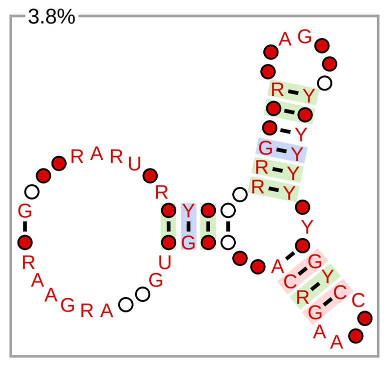

GOLLD-3vp23
subfam_weight=0.509434

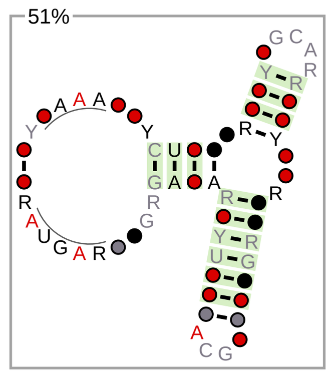

GOLLD-d3classic
subfam_weight=0.245283

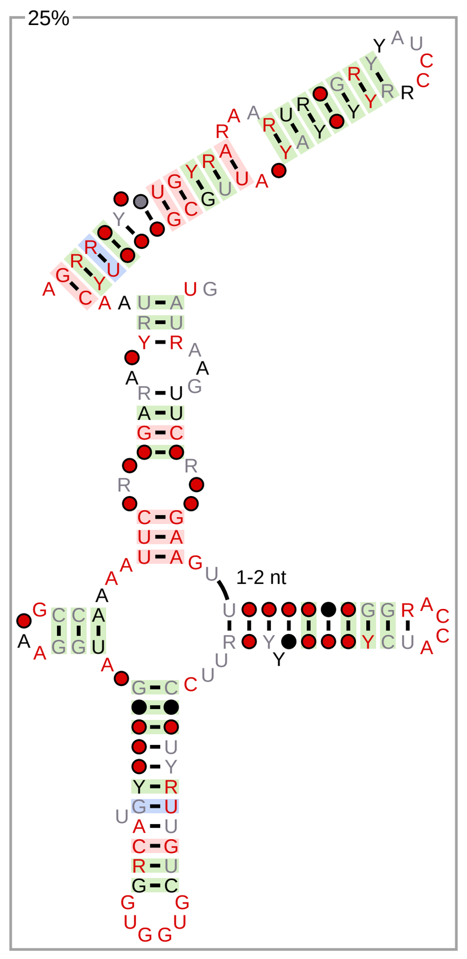

GOLLD-d3justone
subfam_weight=0.132075

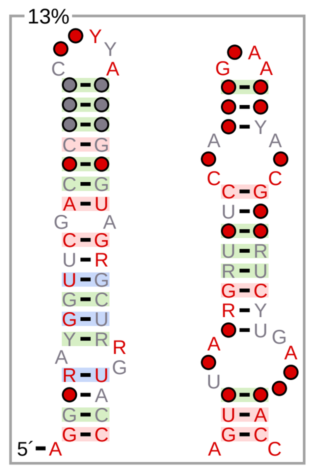

GOLLD-d3valid
subfam_weight=0.377358

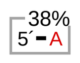

GOLLD-p-1
subfam_weight=0.528302

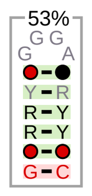

GOLLD-p-1-surround
subfam_weight=0.528302

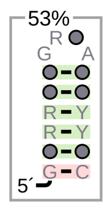

GOLLD-p-2
subfam_weight=0.132075

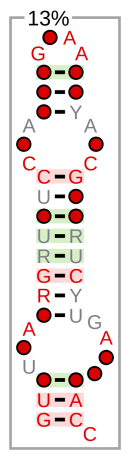

GOLLD-pknot1
subfam_weight=1

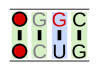

GOLLD-pknot2
subfam_weight=1

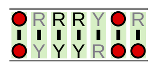

GOLLD-pknot3
subfam_weight=1

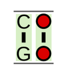

GOLLD-pknot4
subfam_weight=1

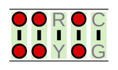

GOLLD-pknot5
subfam_weight=1

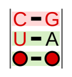

GOLLD-skipbadd2

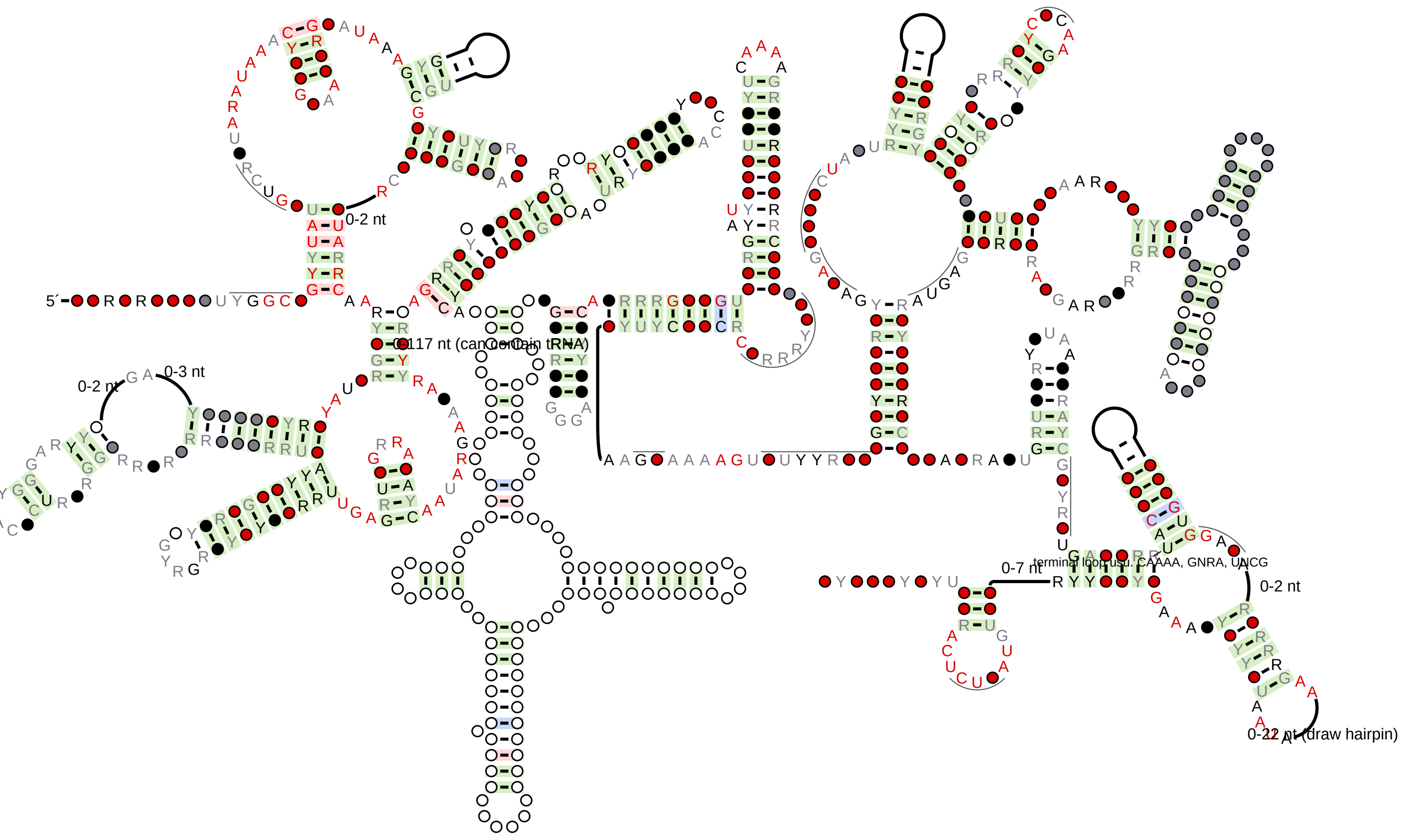

Supplement: Additional file 1 — Source code and example input and output files. C++ and Perl source code is provided. Installation and usage instructions are given in the user manual (Additional file 2), which is also a part of this archive. Example input files for R2R are included in the "demo" subdirectory of this archive, and R2R's raw output on these examples in PDF and SVG format is supplied in the "demo/output-pdf" and "demo/output-svg" subdirectories. Files can be retrieved from the tgz archive file using programs such as WinZip (Windows), StuffIt Expander (Mac) or the tar/gzip commands (UNIX). [file 1471-2105-12-3-S1.TGZ › R2R-1.0.1/demo/output-pdf/GOLLD.pdf]
